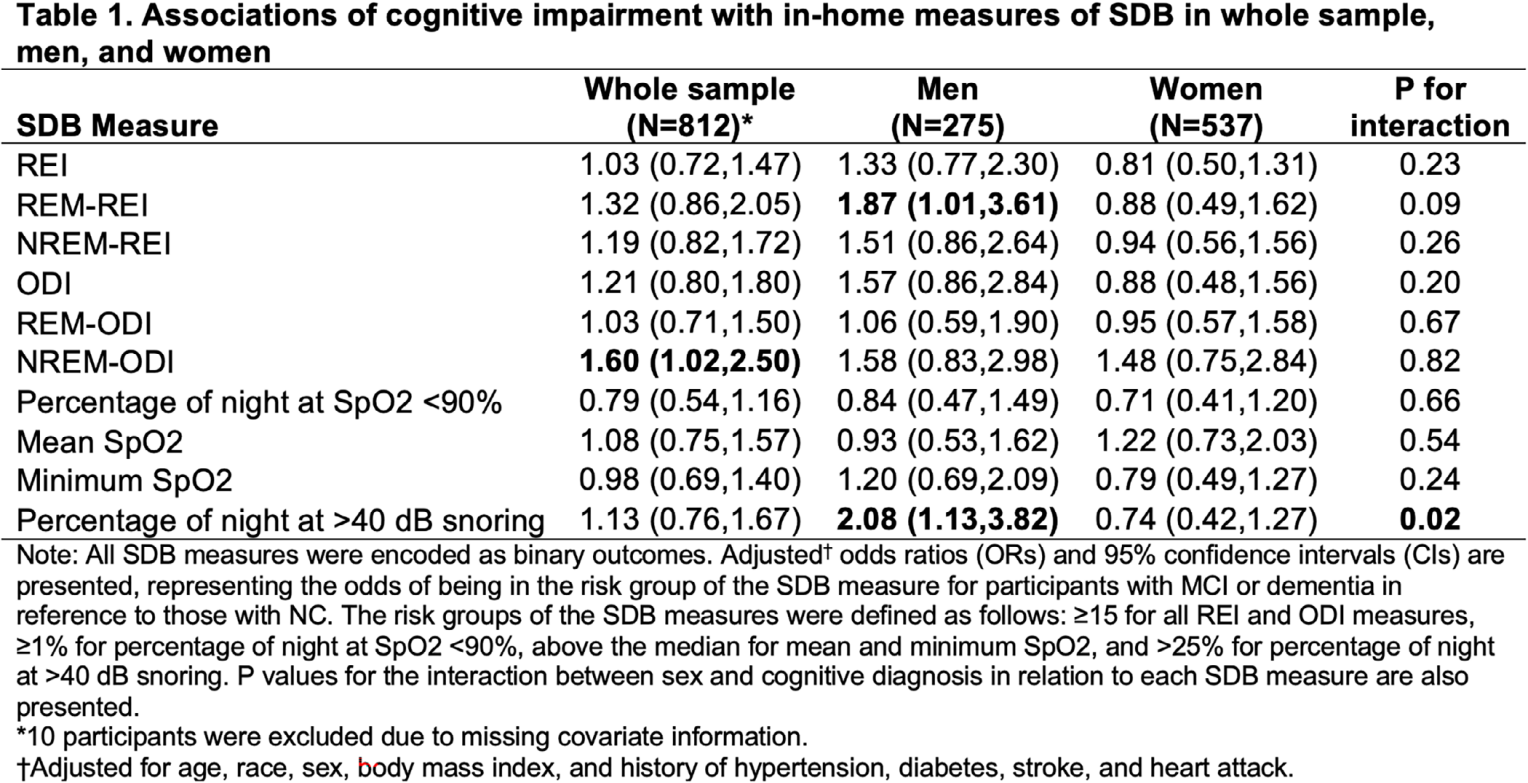# Sex differences in the association of cognitive impairment and sleep‐disordered breathing measures among diverse adults

**DOI:** 10.1002/alz.089902

**Published:** 2025-01-09

**Authors:** Sasha Milton, Carrie B. Peltz, Susan Redline, Sid E. O'Bryant, Kristine Yaffe, Yue Leng

**Affiliations:** ^1^ University of California, San Francisco, San Francisco, CA USA; ^2^ San Francisco Veterans Affairs Health Care System, San Francisco, CA USA; ^3^ Beth Israel Deaconess Medical Center, Boston, MA USA; ^4^ Brigham and Women’s Hospital, Harvard Medical School, Boston, MA USA; ^5^ University of North Texas Health Science Center, Fort Worth, TX USA; ^6^ Departments of Psychiatry and Behavioral Sciences, Neurology, and Epidemiology, University of California San Francisco, San Francisco, CA USA

## Abstract

**Background:**

Growing evidence reports an association between sleep‐disordered breathing (SDB) and cognitive impairment, including mild cognitive impairment (MCI) and dementia. However, there is limited research on the link between cognitive impairment and in‐home measures of SDB and how this association may differ by race, ethnicity, and sex.

**Method:**

We studied 822 individuals who were enrolled in the community‐based Health and Aging Brain Study‐Health Disparities (HABS‐HD)‐Dormir study. In‐home measures of SDB were collected using WatchPAT (WatchPAT‐200). Data were processed using the zzzPAT software (5.2.79.7p) and manually edited by trained polysomnologists. Measures included the respiratory event index (REI), oxygen desaturation index (ODI), snoring volume (percentage of the night over 40 dB), and oxygen saturation (SpO2; three measures, including percentage of night at SpO2 <90%, mean SpO2, and minimum SpO2). A comprehensive clinical evaluation was used to determine normal cognition (NC), MCI, or dementia.

**Result:**

Our sample (mean [SD] age = 66.6 [8.5] years) was 66.2% women; 34.7% Mexican American and 21.2% Black; and 17.3% MCI and 4.1% dementia. After adjustment for age, race, sex, body mass index (BMI), and comorbidities, participants with MCI/dementia had greater odds of a non‐rapid eye movement (NREM)‐ODI of ≥15 [OR (95% CI) = 1.60 (1.02,2.5)] compared to those with NC. Additionally, men with MCI/dementia were more likely to snore at >40 dB for >25% of the night [OR (95% CI) = 2.08 (1.13,3.82)] and to have a rapid eye movement (REM)‐REI ≥15 [OR (95% CI) = 1.87 (1.01,3.61)] than men with NC, while these associations were not present for women [OR (95% CI), snoring = 0.74 (0.42,1.27), p for interaction = 0.02; REM‐REI: 0.88 (0.49,1.62), 0.09]. Other measures of SDB were not associated with cognitive diagnoses. The associations between cognitive diagnoses and SDB measures were similar across race groups.

**Conclusion:**

Among middle‐aged to older men and women, measures of SDB varied in their association with MCI or dementia by sleep stage and by sex. Future investigations should consider sleep‐state specific sleep disturbances as markers or risk factors for cognitive impairment and examine potential sex differences in those associations.